# A Genetic and Functional Relationship between T Cells and Cellular Proliferation in the Adult Hippocampus

**DOI:** 10.1371/journal.pbio.1000561

**Published:** 2010-12-14

**Authors:** Guo-Jen Huang, Adrian L. Smith, Daniel H.D. Gray, Cormac Cosgrove, Benjamin H. Singer, Andrew Edwards, Stuart Sim, Jack M. Parent, Alyssa Johnsen, Richard Mott, Diane Mathis, Paul Klenerman, Christophe Benoist, Jonathan Flint

**Affiliations:** 1 Wellcome Trust Centre for Human Genetics, University of Oxford, Oxford, United Kingdom; 2 Department of Zoology, University of Oxford, Oxford, United Kingdom; 3 Section on Immunology and Immunogenetics, Joslin Diabetes Center and Department of Pathology, Harvard Medical School, Boston, Massachusetts, United States of America; 4 Peter Medawar Building for Pathogen Research, Nuffield Department of Medicine, University of Oxford, Oxford, United Kingdom; 5 Department of Neurology, University of Michigan, Ann Arbor, Michigan, United States of America; Stanford University, United States of America

## Abstract

Neurogenesis continues through the adult life of mice in the subgranular zone of the dentate gyrus in the hippocampus, but its function remains unclear. Measuring cellular proliferation in the hippocampus of 719 outbred heterogeneous stock mice revealed a highly significant correlation with the proportions of CD8+ versus CD4+ T lymphocyte subsets. This correlation reflected shared genetic loci, with the exception of the H-2Ea locus that had a dominant influence on T cell subsets but no impact on neurogenesis. Analysis of knockouts and repopulation of TCRα-deficient mice by subsets of T cells confirmed the influence of T cells on adult neurogenesis, indicating that CD4+ T cells or subpopulations thereof mediate the effect. Our results reveal an organismal impact, broader than hitherto suspected, of the natural genetic variation that controls T cell development and homeostasis.

## Abstract 2

A large correlation between variation in T cell subsets and hippocampal neurogenesis suggests that the immune system has an unexpectedly large influence on the brain.

## Author Summary

In adult mice new neurons are produced in the hippocampus, where they are thought to influence learning, memory, and emotional regulation. The mechanisms and functions of this neurogenesis, however, remain unclear. Here we report that in different strains of mice, variation in cellular proliferation in the hippocampus (an index of neurogenesis) correlates with variation in the relative proportions of the ratio of CD4+ to CD8+ T cells (an immunology phenotype). We also show that T cells can influence neurogenesis (but that neurogenesis does not influence T cells) by analyzing knockouts, depleting mice of T cells, and repopulating alymphoid animals. The strong genetic correlation between T cells and cellular proliferation in the hippocampus contrasts with the weak, often non-significant, correlation with behavioral phenotypes. Of significance, the findings here suggest that modulation of the functions of the hippocampus to influence behavior is not the primary role of neurogenesis.

## Introduction

The discovery that neurogenesis occurs in the adult hippocampus has attracted considerable attention, yet its function remains unclear [1],[2]. Adult neurogenesis is known to occur in two areas of the mammalian brain, the subventricular zone, which gives rise to olfactory bulb interneurons, and the dentate gyrus of the hippocampal formation, which gives rise to granule cells [3]. Its occurrence in the latter structure has prompted considerable speculation that it is involved in known functions of the hippocampus: learning, memory, and emotional regulation. However, the hippocampal function of adult neurogenesis is still debated. Experiments using antimitotic agents and irradiation to kill newly dividing cells in the brain have produced conflicting results (reviewed in [4]). Genetically targeted ablation of neurogenesis also reports contrasting effects: normal learning and memory [5], normal anxiety with a reduction in contextual freezing and normal spatial memory [6], no change in freezing but impaired spatial memory [7],[8], a combined impairment of spatial memory and reduction of contextual freezing responses [9],[10], or increased anxiety but no effect on spatial memory [11].

While it is possible that the behavioural effects of neurogenesis ablation are subtle (a recent report argues that they include specific impairments in spatial discrimination [1]), it may also be the case that adult neurogenesis has additional roles. Here we adopted a genetic approach to address this question. Rates of adult hippocampal neurogenesis differ between inbred strains of mice, indicating that quantitative trait loci (QTL) contribute to this variation [12]. We asked if QTLs influencing neurogenesis could be found that influenced other phenotypes, which might cast light on the function of neurogenesis.

We decided to map variation in adult hippocampal neurogenesis in heterogeneous stock (HS) mice, a stock descended from eight inbred progenitor strains (A/J, AKR/J, BALB/cJ, C3H/HeJ, C57BL/6J, CBA/J, DBA/2J, and LP/J) and maintained for over 50 generations [13]. The large number of recombinants that have accumulated since the founding of the stock means that QTLs are mapped to an average region of 3 Mb, so that co-localization is more likely to reflect pleiotropic action than co-incident location. The HS is unique not only for its high resolution and the number of QTLs that have been mapped (843) [13] but also for the diversity of traits analysed, including disease models (asthma, anxiety, and type 2 diabetes), as well as haematological, immunological, biochemical, and anatomical assays. The phenotypes include those previously suggested to be related to neurogenesis: novelty suppressed feeding [14], measures of anxiety taken in an elevated plus maze and open field, and contextual fear conditioning (data are freely available from http://gscan.well.ox.ac.uk) [9]–[11]. Our aim was to explore the relationship of these, and other, phenotypes to adult neurogenesis in the HS mice.

## Results

### Variation in KI67 Counts Correlates with T Cell Phenotypes

**Figure 1:**
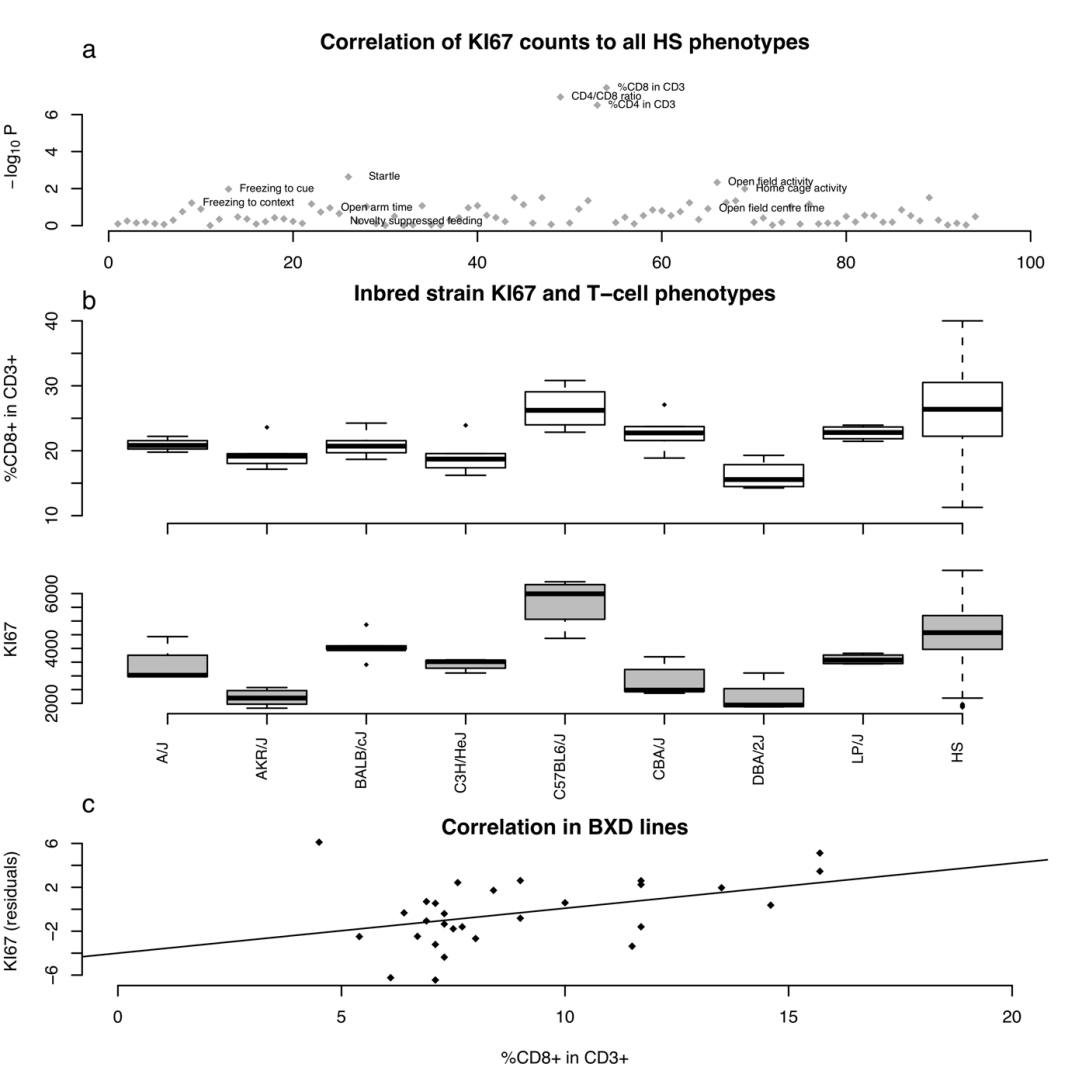
Correlations between hippocampal neurogenesis assayed by counting KI67 positive cells and phenotypes in three populations of mice. (a) Correlations between hippocampal neurogenesis assayed by counting KI67 positive cells and phenotypes measured in outbred mice. Correlations are shown for all 97 phenotypes measured with the negative logarithm of the *p* value (log_10_P) of the Spearman correlation coefficient on the vertical axis. Three T cell measures are labelled, as are eight behavioural measures putatively related to neurogenesis. (b) Boxplot of the distribution of counts of the percentage of CD8+ in CD3+ T cells and KI67 positive cells in the hippocampus in the eight progenitor strains of the HS (A/J, AKR/J, BALB/cJ, C3H/HeJ, C57BL/6J, CDA/J, DBA/2J, and LP/J [16]) and the HS animals themselves. Five animals from each strain were assayed. (c) Scatter plot showing the correlation between KI67 counts and CD8+ in CD3+ T cells in 30 recombinant inbred BXD lines (data from published sources [12],[15]). The line is the least square regression line.

We assessed cellular proliferation in the subgranular zone of the dentate gyrus by counting the absolute number of KI67-positive cells in 719 HS animals (KI67 is a marker of cell proliferation). The phenotypic distribution is shown in Figure 1a. Proliferation correlated with four behavioural measures assessed in the same HS animals. Correlation with one measure in the open-field arena, total activity, was significant (*p* = 0.006) and accounted for just over 1% of the variance. Higher KI67 counts were associated with lower activity levels (since activity increases neurogenesis, the correlation is unlikely to be consequent on the animal's behaviour). A similar pattern was seen in the elevated plus maze: there was a small correlation with distance travelled in the open arms (anxiogenic regions) of the maze (*p* = 0.03, variance explained 0.5%). Proliferation was associated significantly with startle response (*p* = 0.002, variance explained 1.5) and positively correlated with home cage activity (*p* = 0.005, variance explained 1.2%), contrasting with the negative correlation seen for fear-related activity. There was no significant correlation with novelty suppressed feeding (*p* = 0.64), freezing to the context (*p* = 0.09), or freezing to the cue (*p* = 0.92).

We next asked whether cellular proliferation in the hippocampus was correlated with any of the other phenotypes measured in our HS screen, which were a priori unrelated to neurogenesis. Most unexpectedly, we found a highly significant positive correlation with changes in the relative proportions of CD4+ and CD8+ cells among blood CD3+ lymphocytes (*p* = 1.6E−07). Higher levels of hippocampal proliferation are associated with a higher proportion of CD8+ T cells and correspondingly with lower proportions of CD4+ T cells and a lower CD4+/CD8+ ratio. The correlation accounts for about 6% of the phenotypic variance. As Figure 1a shows, the correlation with T cell phenotypes is far more significant than with any other phenotype. The negative logarithm of the *p* value (logP) exceeds a conservative threshold corrected for multiple testing and could not be attributed to the effects of outliers or distributional artefacts (a non-parametric test of rank correlation is also significant).

The same relationship was seen in analyses of inbred strains. Significant correlations were observed between KI67 counts and %CD8 in CD3+ blood T cells (*r* = 0.115, *p* = 0.0008) in the eight inbred progenitor strains of the HS (Figure 1b). Using published data from a large genetic reference panel of recombinant inbred strains (BXD) [12],[15], we found the same correlations between cellular proliferation in the hippocampus and CD8+ T cell frequency (*r* = 0.61, *p* = 0.0006) (Figure 1c). Replication in the two inbred samples provides conclusive evidence for the robustness of the correlation.

### KI67 and T Cell Subpopulation Phenotypes Are Genetically Correlated

The striking correlation between QTLs influencing cellular proliferation in the hippocampus and variation in the distribution of T cell lineages suggests that one phenotype influences the other. We used a genetic approach to address this question and first investigated the extent to which the phenotypic correlation arises from common genetic determinants.

We mapped QTLs contributing to variation in cellular proliferation in the hippocampus [16] and compared their location to QTLs previously mapped for %CD8+ among CD3+ T cells [13]. Table 1 gives a resample-based model inclusion probability (RMIP) [16] for QTLs influencing these phenotypes. The mapping method identifies loci by the number of times they recur in multiple analyses of subsamples of the complete data. From simulations in the HS, a detected QTL that exceeds an RMIP threshold of 0.5 will be true in 85% of cases and in 70% of cases for a threshold of 0.25 [13].

**Table 1:** QTLs that contribute to cellular proliferation in the hippocampus (KI67) and to %CD8 in CD3 T-cells.

Phenotype	Chr	Start	End	RMIP
%CD8+ in CD3	1	59.2	59.9	0.31
KI67	1	132.7	137.7	0.4
%CD8+ in CD3	3	126.9	129.5	0.59
KI67	4	40.4	49.8	0.26
**KI67**	**5**	**71.2**	**76.8**	**0.68**
**%CD8+ in CD3**	**5**	**70.8**	**76.8**	**0.2**
%CD8+ in CD3	5	113.6	114.7	0.94
**KI67**	**6**	**68.8**	**74.8**	**0.22**
**%CD8+ in CD3**	**6**	**68.8**	**73.7**	**0.94**
KI67	6	127.1	127.8	0.27
%CD8+ in CD3	7	47.9	51.8	0.25
%CD8+ in CD3	7	118.4	122.6	0.36
**%CD8+ in CD3**	**10**	**38.3**	**40.3**	**0.2**
**KI67**	**10**	**38.3**	**45.3**	**0.26**
%CD8+ in CD3	12	26.6	30.9	0.77
**KI67**	**13**	**35.2**	**37.7**	**0.53**
**%CD8+ in CD3**	**13**	**36.1**	**37.7**	**0.35**
KI67	13	110.6	119.2	0.49
**KI67**	**16**	**34.4**	**35.7**	**0.67**
**%CD8+ in CD3**	**16**	**34.8**	**36.6**	**0.3**
%CD8+ in CD3	17	33.7	34.5	1
%CD8+ in CD3	17	43.6	49.6	0.58

The table gives the start and end coordinates of the 95% confidence intervals of each QTL (these coordinates are in megabases and are from Build 37 of the mouse genome). Entries in bold indicate QTLs where there is overlap between the two phenotypes. Our measure of probability that the locus is correctly identified is a resample model inclusion probability (RMIP) in the last column. An RMIP is the expected proportion of times a locus is included in a multilocus model. A value of 1 means that the locus would be included in all repeated analyses (estimated by re-sampling the data) and an RMIP of 0.5 means the locus is included in 50% of such analyses. Assessing false positive rates by simulation indicates that at an RMIP threshold of 0.25 about one false positive QTL occurs every four genome scans.

**Figure 2:**
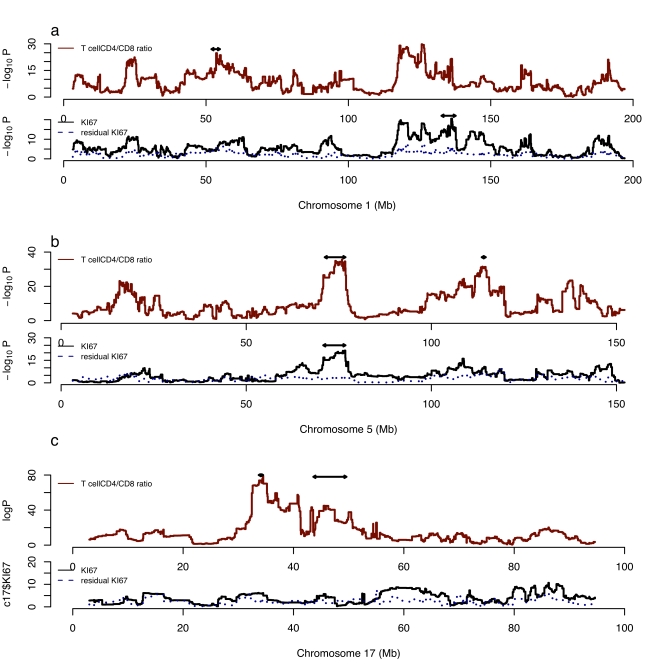
Genetic mapping of cellular proliferation in the hippocampus (KI67), the %CD8+ in CD3 T cells, and the residuals of a model including %CD8+ in CD3 as a covariate (residual KI67). The horizontal scale is the megabase (Mb) distance along the chromosome (build 37 of the mouse genome) and the horizontal scale the negative logarithm of the *p* value (logP) for association between the phenotype and genotype. Results from chromosomes 1, 5, and 17 are shown. Double-headed arrows indicate peaks with a RMIP greater than 0.25. The dotted line in the KI67 plots is the result of mapping the KI67 counts conditioning on %CD8+ in CD3 T cells.

QTLs with the same location for both phenotypes are found on five chromosomes, 5, 6, 10, 13, and 16. However, plots of the logPs frequently identify the same peaks for both phenotypes (Figure 2), suggesting that there may be additional peaks that influence both phenotypes. To test whether variation in T cell lineages and cellular proliferation in the hippocampus have a common genetic origin, we included %CD8+ in CD3+ as a covariate in a linear model for KI67 and mapped the residuals. For each locus we found a marked reduction in the significance of the association for KI67. This is illustrated in Figure 2, where it is apparent for example on chromosome 5 that the logP drops from more than 15 to less than 3. Similar results were obtained when we mapped %CD8+ in CD3+ including KI67 counts as a covariate. These results indicate that common genetic loci contribute to variation in the two phenotypes.

There is also evidence for loci with specific effects: the single largest QTL effect on CD4+ versus CD8+ T cell subsets scarcely contributes to KI67 counts. This latter QTL lies on chromosome 17 within the Major Histocompatibility Complex (MHC) and is consistent with a strong impact of variation in the MHC in determining the relative distribution of CD4+ and CD8+ cells [17],[18]. The QTL arises from a deletion of the promoter of H2-Ea [17] that encodes the alpha chain of the MHC class II Eαβ heterodimer, one of the two complexes that govern the selection and survival of CD4^+^ T cells. In the HS this QTL has an RMIP of 1 for CD4+ or CD8+ T cells (logP>80) but is excluded from all re-sampled multiple QTL models influencing KI67 counts (logP<4) (Figure 2).

Comparison between QTLs influencing behavioural measures putatively related to hippocampal neurogenesis and KI67 showed little overlap in location (results for all phenotypes are presented online at http://gscan.well.ox.ac.uk). We quantified this relationship by exploiting the fact that QTLs detected in the HS explain about 70% of the heritability of each phenotype [13]. Consequently the extent of overlap in QTL location is a measure of genetic correlation.

**Figure 3:**
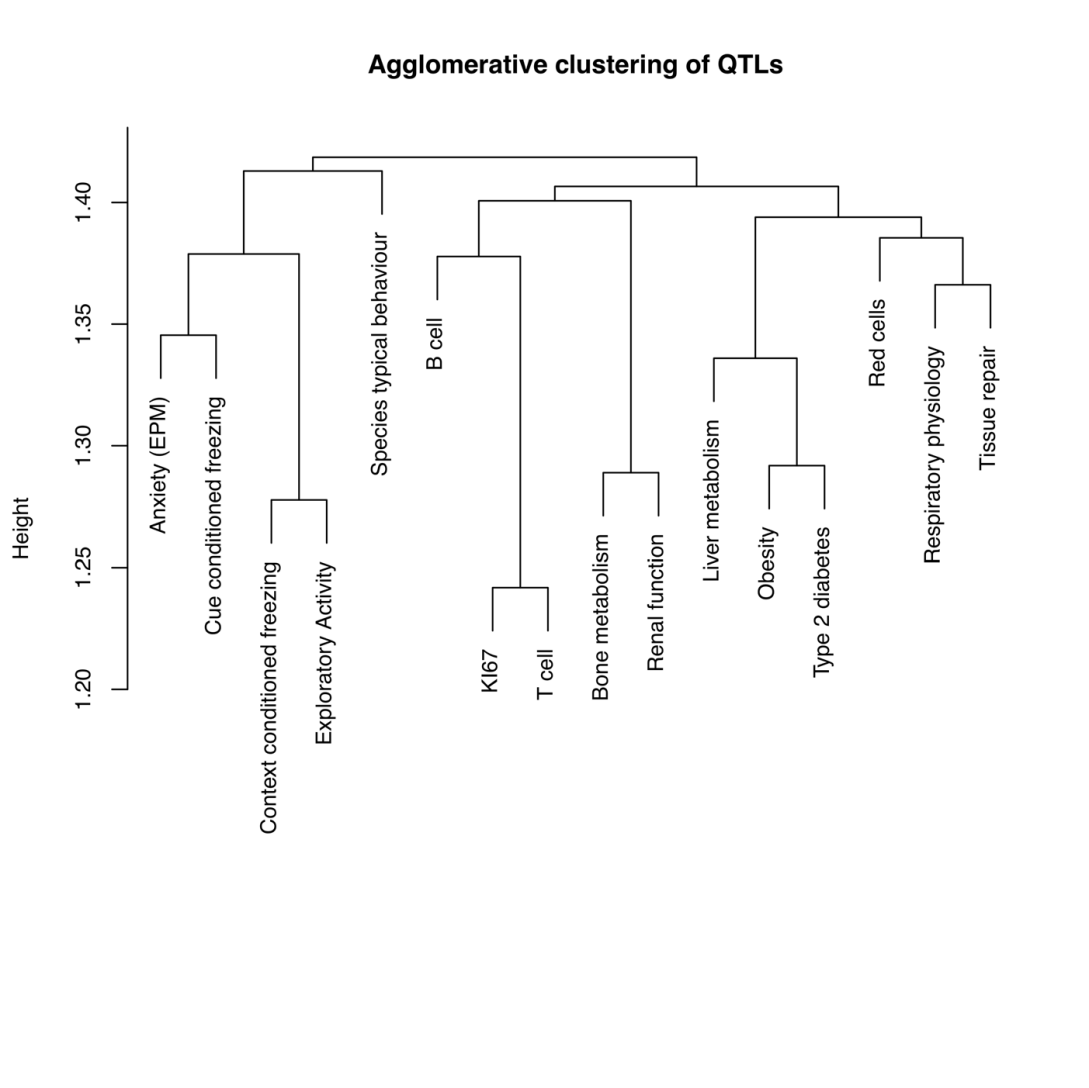
Agglomerative clustering of a genetic distance matrix to show the relationship between cellular proliferation in the hippocampus (KI67), behaviour, and other phenotypes obtained in the heterogeneous stock mice. The distance matrix is based on the degree of QTL overlap. In order to simplify the presentation, the 97 phenotypes were grouped into 16 trait sets according to the disease models that they were designed to test, or on their physiological functions (for instance, combining all QTLs for T cell function or for hippocampal function). Species typical behaviour, burrowing, has been shown to be affected by hippocampal lesions yet does not reflect emotional reactivity [48].

For each pair of phenotypes we calculated the total length of the QTLs and the total length of the overlap and used a distance measure (Sørensen's similarity coefficient) to construct a tree of the relationships between phenotypes (using a clustering algorithm). Analysis of all 97 individual phenotypes shows that cellular proliferation in the hippocampus is genetically most closely related to variation in T cell subsets. We show this result in Figure 3, where we have simplified the presentation of 97 phenotypes by grouping them into 16 trait sets according to the disease models that they were designed to test, or on their physiological functions. Phenotypic data are available from http://gscan.well.ox.ac.uk.

### CD4+ T Cells Mediate the Effect on Adult Neurogenesis

Our genetic data are consistent with a model in which adult neurogenesis influences T cells and also with one in which T cells influence neurogenesis (where our measure of neurogenesis is cellular proliferation in the hippocampus). We tested the first model by ablating adult neurogenesis and then looking for changes in T cells. We did this using i.c.v. ganciclovir (GCV) in both GFAP-tk [6] and nestin-tk mice [19]. Four weeks later, we collected blood samples and quantitated CD4+ and CD8+ T cell subsets. This schedule is effective in ablating dividing putative forebrain progenitors in GFAP-TK mice while avoiding the gut illness caused by high doses of GCV [20],[21]. The hippocampus was stained with a marker for immature neurons (doublecortin, DCX). DCX positive cells were depleted in both models. Compared to untreated littermates we found no significant difference in T cell subsets after ablation of neurogenesis in either model (GFAP-tk: *t* = −1.7, *df* = 14, *p* value = 0.1; nestin-tk: *t* = 0.1, *df* = 14, *p* = 0.9).

**Figure 4:**
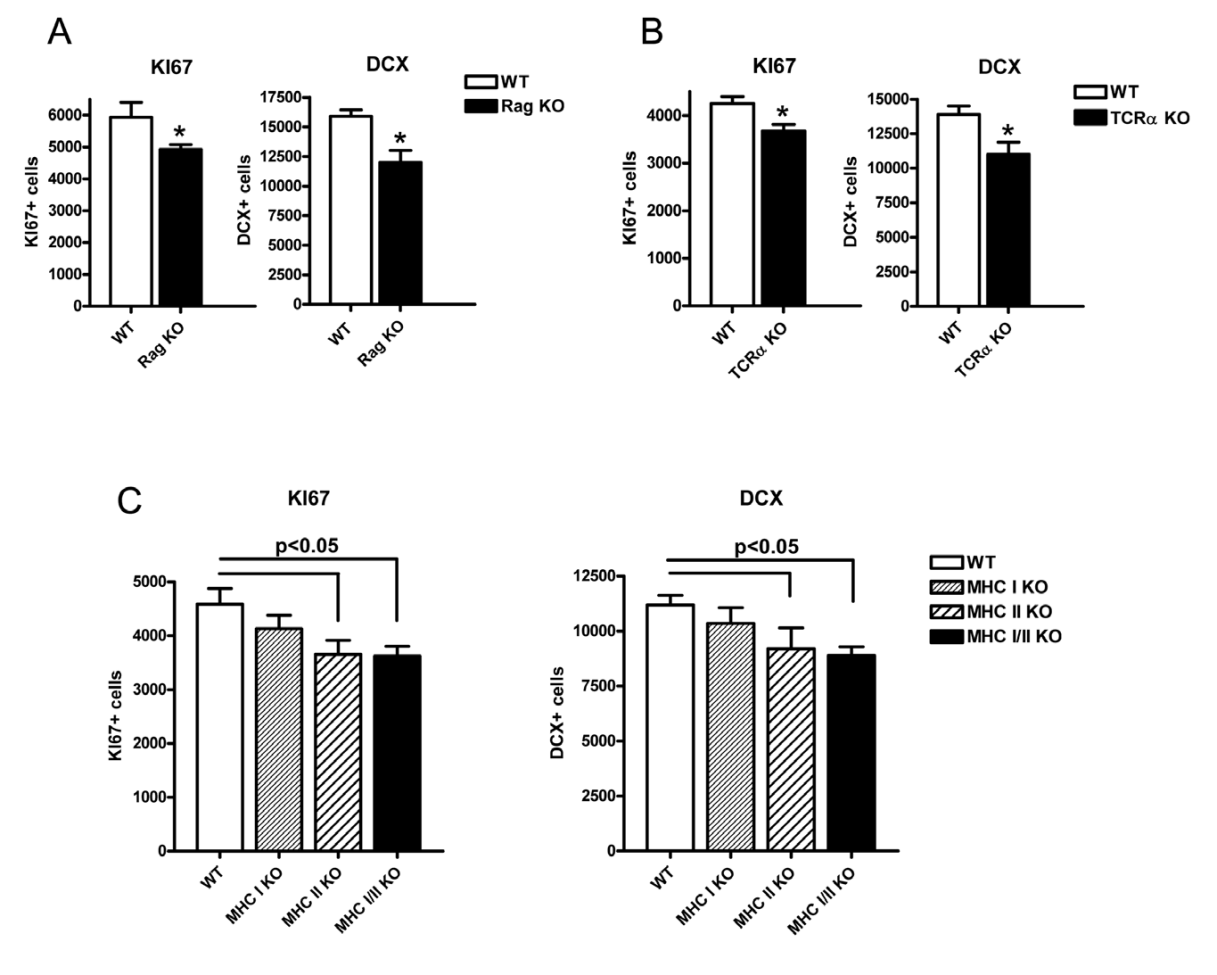
Effect of T cell knockouts on adult neurogenesis. (A) Effect of RAG knockout and corresponding littermate wild types (*n* = 5) on KI67 and doublecortin (DCX) counts in the dentate gyrus of the hippocampus. (B) Effect of TCRα knockout and corresponding littermate wild types (*n* = 7) on KI67 and doublecortin (DCX) counts in the dentate gyrus of the hippocampus. Each group consisted of seven animals. (C) Effect of MHC Class I (*n* = 9), Class II (*n* = 5), and combined knockouts (*n* = 7) and corresponding littermate wild types (*n* = 8) on KI67 and DCX counts in the dentate gyrus of the hippocampus. Values shown are means ± SEM. * *p*<0.05.

We tested the second hypothesis, an effect of T cell function on neurogenesis, by examining a series of mouse mutants with alterations in T cell differentiation. As well as the cell proliferation marker KI67, we used DCX to detect hippocampal neurogenesis in 10-wk-old mice. We first investigated Rag-deficient mice, which are devoid of any B or T lymphocytes: mutant mice showed a marked reduction in both readouts of neurogenesis (KI67 and DCX) (Figure 4A; *p* = 0.02 (KI67) and *p* = 0.01 (DCX)). This was also true for TCRα knockouts (Figure 4B, *p* = 0.01 (KI67) and *p* = 0.02 (DCX)), implicating cells expressing the αβT cell receptor and excluding B and γδT lymphocytes.

To determine which of the CD4+ or CD8+ lineages of αβT cells had the most influence, we tested knockout mice affecting MHC-I or MHC-II molecules (which are largely devoid of CD8+ and CD4+ T cells, respectively) as well as double-knockout animals. A reduction in KI67 and DCX was present for both MHC-II (*p* = 0.03 (KI67) and *p* = 0.04 (DCX)) and MHC-I/II deficient mice (*p* = 0.006 (KI67) and *p* = 0.002 (DCX)), suggesting that the presence of CD4+ cells mediate the effect of lymphocytes on neurogenesis (Figure 4C).

**Figure 5:**
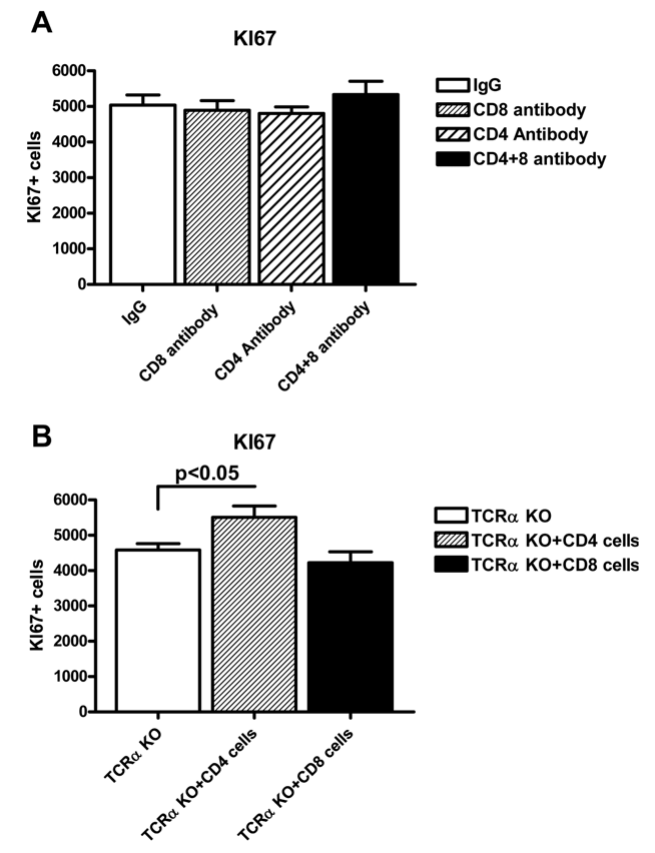
Effect of depletion and repopulation of T cells on adult neurogenesis. (A) The KI67 positive cell counts in the dentate gyrus of the hippocampus 10 d after CD4, CD8, or CD4 and CD8 T cell depletion by antibody injection. Each group consisted of eight animals. (B) The KI67 positive cell counts in the hippocampus after 2 wk of reconstitution of TCRα knockout mice with CD4 or CD8 lymphocytes by intraperitoneal injection of 2×10^7^ cells/mouse. Each group consisted of six animals. Values shown are means ± SEM.

We performed two independent tests of the functional involvement of CD4+ and CD8+ T cells. First, we depleted either population by injection of anti-CD4 or anti-CD8 monoclonal antibodies, reducing their numbers to less than 5% of untreated mice at 10 d post-treatment. Despite the successful depletion of T-cell subsets, there was no significant change of KI67 levels in the dentate gyrus of the hippocampus (Figure 5A). Second, we performed a repopulation experiment, in which 2×10^7^ CD4+ or CD8+ T cells from spleen and lymph node were transferred into TCRα-deficient hosts. Transfer of CD4+ T cells led to a significant increase in KI67 staining in 2 wk post-transfer (compared to the TCRα knockout *p* = 0.03). In contrast transfer of CD8+ T cells did not alter KI67 staining (Figure 5B).

## Discussion

Our analysis of a large stock of outbred mice makes two novel observations about adult neurogenesis in the hippocampus. First, we find a correlation between variation in cellular proliferation in the hippocampus and the relative proportions of CD4+ and CD8+ subsets of αβT cells. The correlation is much larger than that found for over 90 other phenotypes. Second, the correlation is driven by genetics: QTLs that contribute to variation in cell proliferation in the hippocampus also contribute to variation in the relative proportions of CD4+ and CD8+ T cells. Importantly, the genetic correlation is due to natural sequence variants that differentiate inbred strains of laboratory mice.

Our findings extend previous observations that T cells contribute to variation in adult neurogenesis [22],[23]. Previously, analysis of mice with a mutation that ablated both T- and B-lymphocyte compartments showed impairment of adult neurogenesis, which could be restored by repopulation with mono-specific T cells recognizing a CNS-antigen (myelin basic-protein). This was interpreted as an effect of autoimmune attack [22]. Reduced adult neurogenesis was also found in mice with no T or B cells due to a mutation in either RAG1 or RAG2 [23]. Repopulation and depletion experiments again implicated T cells [23]. The latter experiments, together with analysis of CD4−/− mice, indicated that CD4+ T cells contributed to adult neurogenesis, regardless of their antigen specificity.

Results obtained from the HS indicate that the relationship between T cells and cellular proliferation in the hippocampus is not merely an artefact of transgenic or knockout mice, or of the very unusual conditions created in alymphoid mice upon immune reconstitution, but arises from the far more subtle cues of natural genetic variation: loci that contribute to variation in cell proliferation in the hippocampus also contribute to variation in the relative proportions of CD4+ and CD8+ T cells. Naturally occurring genetic variation is a very different setting (and obviously more physiogically relevant) than the gross abnormalities of knockout mice.

Two questions follow: why do T cells influence neurogenesis in the hippocampus, and how do they do it? Our data suggest a broader role for T cell function on adult neurogenesis than previously suspected [22],[23]. The strikingly large correlation between cellular proliferation in the hippocampus and T cell subsets, attributable to naturally occurring genetic variation, suggests that the correlation itself is functionally important (otherwise it would have decayed through stochastic fluctuation in allele frequencies). The strong correlation contrasts with the weak, often non-significant, correlation with behavioural phenotypes often invoked as functional consequences of adult neurogenesis. This suggests that modulation of the behavioural functions of the hippocampus may not be the primary role of neurogenesis. Postulated roles in learning and memory may in fact be secondary to the immunological [1],[2]. For example, anything that alters the ratio of CD4+ to CD8+ cells might alter behaviour, so that variation in behavioural results from different laboratories [1],[2] could be due to differences in the health status of the animals. It is also interesting to note alterations in CD4+ lymphocytes may affect human behaviour. For example, the mechanism by which human immunodeficiency virus type 1 (HIV-1) produces dementia remains obscure [24]. Impairment of neurogenesis is suspected to play a part [25],[26] and this may be mediated in part by reduction in CD4+ lymphocytes.

How do T cells influence neurogenesis in the hippocampus? This is difficult to explain, given that T cells are very rare in the brain under normal circumstances. Moreover genetic mapping experiments, and those performed in knockout or reconstituted mice, appear to give different clues about mechanism: analysis of the HS mice pointed to the involvement of CD8+ T cells, as their frequency correlated with increased neurogenesis, while the analysis of knockouts and repopulation experiments implicated CD4+ T cells, here and in the work of Wolf et al. [23].

An important clue comes from the delineation of the QTLs that affect both T cell and neurogenesis phenotypes. All QTLs that control the proportions of CD4+ and CD8+ T cells also have an impact on neurogenesis, with the glaring exception of the strongest of them, the H2-Ea locus [17], whose homolog has also been identified in human GWAS studies of CD4+/CD8+ ratio determinism [18]. H2-Ea allows the assembly of a functional EαEβ MHC-II heterodimer. In turn, this E heterodimer, by presenting an array of self-peptides, can select and support a large population of “E-restricted” CD4+ T cells. H2-Ea is frequently inactivated by deletions or other mutations that are widespread among inbred and wild mice [27],[28], and such mice (including the C57BL/6 founder of the HS stock) only have the AαAβ MHC-II complex and A-restricted CD4+ T cells. E-restricted CD4+ T cells are functionally effective by all measures tested [29], but the strong protective effect of H-2Ea against autoimmune diseases such as Type-1 diabetes suggests that E- and A-restricted repertoires are not always equivalent. In the present context, the absence of a neurogenesis QTL mapping to H-2Ea suggests that the connection between CD4+ T cells and neurogenesis is a property that can be uniquely fulfilled by A-restricted cells, implying a role for specific TCR/MHC interactions, rather than a generic effect of the relative proportions of CD4+ and CD8+ T cells.

In regards to the apparent discrepancy between the impact of natural genetic variation and the results of experimental manipulation, it is important to realize that different populations are being affected in the different modalities. CD4+ T cells are a complex mix of cells. Some of them have positive effector functions, while others exhibit regulatory potential over other cells of the immune system and also over non-lymphoid organs such as the adipose tissue (in particular FoxP3+ “Treg” cells [30],[31]). While natural variation might equally affect all CD4+ T cells, the conditions created by the lineage deficiency of MHC knockouts, which still have a significant Treg component, or by the homeostasis-driven proliferation that occurs after reconstitution of lymphopenic hosts [32] may result in an altered balance of effector versus regulatory T cells. Thus, the same subpopulations of CD4+ T cells are not equally affected in all instances, and a unifying interpretation may be that neurogenesis positively correlates with the relative proportion of CD4+ and CD8+ effector relative to Treg cells.

In cellular terms, how A-restricted effector or regulatory T-cells influence hippocampal activity remains conjectural. One might hypothesize direct interactions occurring in the hippocampus between CD4+ T cells and MHC-II+ microglia. CD4+ T cells are very rare but not completely absent in this anatomical location. Alternatively, the hippocampus may be responding to long-range mediators released by T cells, for instance the cytokines released during T cell activation in lymphoid organs.

One candidate for the molecular mechanism set in motion by T cells is the Wnt pathway [33],[34]. Wnt signalling components are expressed in adult hippocampal progenitor cells and modulate the generation of newborn neurons [35]. Inhibition of Wnt signalling increases the number of new neurons and leads to a loss of stem cell multipotency [36]. Manipulation of recently identified components of the Wnt pathway (NEUROD1 [37],[38] and DISC1 [39]) also alters the production of new neurons. However, mediators between T cells and the Wnt pathway are unknown.

We expect clues to their identity to emerge from identifying the molecules under the QTLs that co-regulate T cells and cellular proliferation in the hippocampus. Mapping resolution in the HS is approximately 3 Mb, insufficient to formally identify single genes. However, it is worth noting that the locus on chromosome 6 contains the *Cd8a* and *Cd8b* genes, whose products are expressed in CD4+CD8+ immature precursors in the thymus, and would be expected to influence lineage commitment to either the CD4+ or CD8+ lineages. A locus on chromosome 13 contains *Fst* (Follistatin), already known to be involved in adult neurogenesis [40].

The interaction between neurogenesis and T cell subsets is another example of neural-immune communication across the blood brain barrier: it is now clear that this happens outside of disease states [41] and that a common molecular machinery may operate in neurons and immune cells. Major histocompatibility molecules and parts of the complement cascade are involved in neural development and function [42]; many of the same cell adhesion molecules regulate the specificity of interactions at both neuronal and immunological synapses [43].

Our insights into the role of T cells in adult neurogenesis depended upon access to multiple phenotypes obtained in a set of genotyped animals. The delineation of biological control pathways is hard to achieve through experiments that test one phenotype, and one gene, at a time. The approach used here is likely to become more common with the availability of genetic reference populations, such as the collaborative cross [44],[45].

## Methods

### Animals

Original Northport HS mice were obtained from Dr. Robert Hitzemann at the Oregon Health Sciences Unit. Inbred strains (A/J, AKR/J, BALB/cJ, C3H/HeJ, C57BL/6 J, CBA/J, DBA/2 J, and LP/J) were obtained from Harlan (UK) or the Jackson Laboratory (Bar Harbor, ME). TCRα (Philpott PMID: 1604321), RAG (PMID: 1547488), and MHC (Aβ and β2M, Cosgrove PMID: 1909605 and Koller PMID: 2682666, respectively) deficient mice were from our custom-breeding colony housed at the Jackson; wild-type controls used as comparators were littermates from the same crosses. Neurogenesis parameters were analysed in 10-wk-old animals. All mice were maintained in a specific pathogen free facility under barrier conditions.

### Phenotype Assays in the HS

All behavioural and other assays performed in the HS animal are described in [46].

### Inducible Ablation of Adult Neurogenesis

Ablation of adult neurogenesis was performed with both cellular and temporal specificity using the nestin-tk mouse [19]. The mice express the thymidine kinase (tk) gene from HSV type 1, under the control of a minimal TK promoter element followed by a 1.8 kb fragment of the 2nd intronic nestin enhancer [47]. GFAP-tk mice were obtained from the Jackson Laboratory. We administered GCV via ICV infusion at a rate of 0.25 µl/h by using an osmotic minipump. Minipumps (Alzet, model 2004) were filled with 2 mM GCV and primed for 36 h at 37°C. For implantation of the pump and cannula, mice were anesthetized with 130 mg/kg ketamine and 20 mg/kg xylazine and fixed to a stereotaxic frame after loss of the paw withdrawal reflex. The osmotic pump was implanted subcutaneously over the scapulae and fitted to an intraven-tricular cannula (Brain Infusion Kit 3, Alzet) implanted 0.21 mm anterior, 0.85 mm lateral, and 3.0 mm deep to Bregma. Following surgery, all mice were housed individually for the remainder of the experiment.

### Immunohistochemistry

For KI67 staining, sections were mounted on the superfrost slide (BDH, UK), dried overnight, incubated in the 0.01 mol/L citric buffer for 40 min at 90, incubated in 3% H_2_O_2_ for 10 min, rinsed, and incubated overnight at room temperature with rabbit anti-KI67 antibody (1∶4000, Vector Lab). Next day, a standard rabbit IgG ABC kit procedure was used and reacted 5–10 min with Sigma DAB tablet. Sections were then counterstained with cresyl violet and cover-slipped under DPX. KI67-labeled cells were counted on every eighth section through the entire rostrocaudal extent of the granule cell layer bilaterally. Section thickness is 40 µm. For DCX staining, free floating sections were incubated with goat anti-DCX antibody (1∶400, Santa Cruz Labs), then following the same ABC kit procedure, reacted with Sigma DAB tablet. DCX-labeled cells were counted on every sixteenth section through the entire rostrocaudal extent of the granule cell layer bilaterally. Section thickness is 40 µm.

### Flow Cytometry

Blood samples (100–150 µl) were gathered in EDTA via tail-vein phlebotomy. Erythrocytes were lysed in 5 rounds of incubation with ACK lysis buffer followed by centrifugation. Blocking in Fc solution was followed by incubation with CD3-PE, CD4-APC, and CD8 FITC (eBiosciences) and two rinses in FACS buffer. Flow cytometry was then performed on a BD FacsCanto II instrument with FACS Diva software (BD Biosciences). Counts of CD4 and CD8 positive cells were gated on the CD3 population, and all percentages are expressed as a fraction of CD3+ cells.

### Depletion of T Cell Subsets by Antibody

C57BL6/J mice (*n* = 8 in each group) received mouse received intraperitoneal injections of anti-CD4 (YTS 191 and YTA 3.1.2) or anti-CD8 (169) antibodies or both together, and the control mice were injected with YK1X337 IgG. Antibodies were given over a 3-d period, 0.5 mg per injection of anti-CD8, anti-CD4, or control IgG antibody injection. Antibodies for depletion were a gift from Dr. Stephen P. Cobbold, Sir William Dunn School of Pathology, Oxford, UK. Mice were killed 8 d after the last injection. Blood samples were collected for CD3, CD4, and CD8 analysis.

### T Cell Isolation Transfer

Single cell suspensions of splenocytes from C57BL6/J donors were subjected to magnetic bead cell sorting using anti-CD4 or anti-CD8α paramagnetic beads (Miltenyi-Biotech) and positively sorted with the AutoMACs cell separation equipment (Miltenyi-Biotech) according to the manufacturer's instructions. Briefly, cells were incubated with beads on ice for 20 min in PBS 2 mM EDTA washed and applied to the AutoMACS, and positive and negative fractions were collected and tested for purity prior to transfer (all transferred populations were >96% pure as judged by FACS). Sorted cell populations were washed by centrifugation in DMEM, resuspended to 8×10^7^/ml and administered to TCRα KO mice (*n* = 6 in each group) by intraperitoneal injection of 250 µl/mouse (2×10^7^/mouse).

## Abbreviations

GCV: ganciclovir
HS: heterogeneous stock
MHC: Major Histocompatibility Complex
QTL: quantitative trait loci
RMIP: resample-based model inclusion probability
