# Correction: A Genetic and Functional Relationship between T Cells and Cellular Proliferation in the Adult Hippocampus

**DOI:** 10.1371/annotation/f4594c2b-b835-4e1a-a7d6-8f2e58f60be5

**Published:** 2011-05-25

**Authors:** Guo-Jen Huang, Adrian L. Smith, Daniel H.D. Gray, Cormac Cosgrove, Benjamin H. Singer, Andrew Edwards, Stuart Sim, Jack M. Parent, Alyssa Johnsen, Richard Mott, Diane Mathis, Paul Klenerman, Christophe Benoist, Jonathan Flint

The name of the 7th author was misspelled. The correct spelling is Stuart Sims. 

